# Wearable Cardioverter-Defibrillator in Patients at Transient Risk of Sudden Cardiac Death: State of the Art and Contemporary Clinical Evidence

**DOI:** 10.3390/diagnostics16182990

**Published:** 2026-09-15

**Authors:** Andrea Matteucci, Michela Bonanni, Marco Valerio Mariani, Luca Sgarra, Lorenzo Nesti, Nicola Pierucci, Stefania Angela Di Fusco, Silvio Fedele, Federico Nardi, Furio Colivicchi

**Affiliations:** 1 Clinical and Rehabilitation Cardiology Division, San Filippo Neri Hospital, 00135 Rome, Italy; 2 Division of Cardiology, Tor Vergata University, 00133 Rome, Italy; 3 Department of Cardiovascular, Respiratory, Nephrological, Anesthesiological and Geriatric Sciences, “Sapienza” University of Rome, 00185 Rome, Italy; 4 Department of Cardiology, Regional General Hospital “F.Miulli”, 70021 Bari, Italy; 5 Department of Clinical and Experimental Medicine, Cardiopulmonary Lab, University of Pisa, 56126 Pisa, Italy; lorenzo.nesti@phd.unipi.it; 6 Department of Interventional Cardiology, UOC Cardiologia, Sandro Pertini Hospital, 00157 Rome, Italy; 7 Department of Cardiology, Santo Spirito Hospital, Casale Monferrato, 15033 Alessandria, Italy

**Keywords:** wearable cardioverter-defibrillator, sudden cardiac death, myocardial infarction, cardiomyopathy, heart failure, myocarditis, ventricular arrhythmia, cost-effectiveness

## Abstract

Sudden cardiac death remains a major mode of cardiovascular mortality, yet the risk is neither constant over time nor adequately represented by left ventricular ejection fraction alone. The weeks after myocardial infarction and the early months after a new diagnosis of cardiomyopathy or acute myocarditis create arrhythmic vulnerability, and recovery with revascularization, guideline-directed medical therapy, rhythm control, or resolution of inflammation may render immediate implantable cardioverter-defibrillator implantation unnecessary or inappropriate. The wearable cardioverter-defibrillator (WCD) was developed to bridge this interval by providing non-invasive, continuously available detection and shock therapy for sustained ventricular tachyarrhythmias. The only randomized trial, VEST, did not significantly reduce arrhythmic death in the intention-to-treat analysis, and therefore does not support indiscriminate WCD prescription after myocardial infarction. Conversely, large registries and contemporary meta-analyses consistently document successful termination of malignant ventricular arrhythmias, clustering of events during the first weeks, and frequent subsequent recovery of systolic function. Economic models from several healthcare systems suggest that WCD use may be cost-effective in selected settings, although estimates are highly dependent on assumptions regarding baseline arrhythmic risk, treatment effectiveness, adherence, and healthcare-system costs, and several analyses have incorporated exposure-based VEST estimates or manufacturer involvement. Overall, current evidence supports the technical efficacy of the WCD for terminating sustained ventricular tachyarrhythmias, but observational shock rates do not establish a population-level survival benefit. The most defensible contemporary role of the WCD is therefore as a selective bridge-to-decision strategy in patients with transient or evolving arrhythmic risk, integrating not only LVEF but also substrate, arrhythmic triggers, timing, and clinical trajectory.

## 1. Introduction

Sudden cardiac death (SCD) remains a major cause of cardiovascular mortality in Europe and worldwide, and ventricular tachycardia (VT) or ventricular fibrillation (VF) remains the dominant immediately treatable mechanism in a substantial proportion of cases [1]. The clinical problem is not simply identifying patients with a chronically elevated risk, but identifying those whose risk is high during a short interval in which the myocardium is vulnerable but may still recover. This distinction is particularly relevant after myocardial infarction (MI), after a new diagnosis of heart failure with reduced ejection fraction (HFrEF) or non-ischaemic cardiomyopathy, and during acute myocarditis. After MI with left ventricular dysfunction, SCD risk is highest early and declines as the substrate heals, revascularization takes effect, neurohormonal therapy is introduced, and competing modes of death change [2]. The implantable cardioverter-defibrillator (ICD) is an established therapy for selected patients with persistent long-term risk, but guideline pathways intentionally delay primary-prevention implantation after acute MI and during the initial phase of newly diagnosed systolic dysfunction [3,4]. The 2025 American College of Cardiology/American Heart Association acute coronary syndrome guideline continues to recommend primary-prevention ICD implantation only after the established post-MI and post-revascularization waiting intervals, while stating: “In patients early after MI, usefulness of a temporary wearable cardioverter-defibrillator is uncertain in patients with an LVEF ≤ 35% to improve survival” (Class 2b, level B-R) [5]. In parallel, the 2025 European Society of Cardiology (ESC) myocarditis and pericarditis guideline has given the WCD a more explicit role in inflammatory heart disease, especially when sustained ventricular arrhythmia occurs during the acute phase [6].

The WCD occupies this therapeutic gap. It is an extracorporeal defibrillation system that continuously analyzes surface electrocardiographic signals, warns a conscious patient before therapy, and can deliver high-energy shocks for persistent life-threatening ventricular tachyarrhythmias. Unlike an ICD, it can be prescribed immediately, removed when risk resolves, and used without an intravascular lead or surgical pocket. Professional statements have consequently framed it as a temporary option for selected patients at transient risk or for patients in whom an indicated ICD is temporarily contraindicated [7,8,9]. However, the WCD remains one of the most debated technologies in arrhythmia prevention. Early feasibility studies and registries demonstrated technical efficacy [10,11,12,13,14,15], whereas the only major randomized trial after MI did not meet its primary arrhythmic-death endpoint. Contemporary meta-analyses, including 59,647 patients, report an overall appropriate WCD intervention rate around 3%, and higher event rates during early follow-up [16]. An updated systematic review has emphasized that the randomized evidence remains negative and that observational evidence is inherently vulnerable to selection bias [17], while an earlier meta-analysis similarly found substantial heterogeneity and large differences between observational cohorts and the randomized setting [18]. The central question has therefore changed. Rather than asking whether every patient with a low LVEF should receive a WCD, the relevant question is which transient-risk phenotypes derive enough short-term protection and decision-making value to justify the device.

## 2. Methods and Literature Search Strategy

The literature search was conducted on PubMed, EMBASE and Cochrane Library, from inception through July 2026. Search concepts included “wearable cardioverter defibrillator”, “wearable defibrillator”, “sudden cardiac death”, “ventricular arrhythmia”, “myocardial infarction”, “acute coronary syndrome”, “cardiomyopathy”, “heart failure”, “myocarditis”, “cardiac magnetic resonance”, “late gadolinium enhancement”, “adherence”, “remote monitoring”, “cost-effectiveness”, “cost-utility”, and “health technology assessment”. Priority was given to randomized trials, large prospective or national registries, systematic reviews and meta-analyses, studies specifically addressing the major clinical indications, and economic evaluations from public healthcare perspectives. Guideline documents from the ESC and ACC/AHA were reviewed directly. References of major reviews and the authors’ previous WCD publications were used to identify additional primary studies. No new pooled estimate was generated, and no formal PRISMA flow diagram or de novo risk-of-bias assessment was performed. This approach is deliberate because the clinically relevant literature is heterogeneous in indication, denominator, duration of wear, adjudication of events, comparison groups, and economic assumptions. The review therefore distinguishes technical efficacy (successful treatment of a detected VT/VF episode), effectiveness during actual wear, population-level survival effectiveness, and the broader value of a WCD-guided bridge-to-decision strategy. For quantitative estimates, 95% confidence intervals and *p*-values are reported when provided by the original source; when no conventional measure of uncertainty was reported, the point estimate is reproduced as published.

## 3. WCD Technology and Application

The modern WCD combines four surface ECG sensing electrodes with therapy electrodes incorporated into a garment and connected to a battery-powered monitor. When an algorithm identifies a sustained tachyarrhythmia in the programmed treatment zone, alarms are triggered before shock delivery. A conscious patient can withhold therapy by pressing response buttons, an important safety feature for reducing inappropriate treatment of artifact or tolerated rhythm. If the patient becomes unresponsive, therapy is delivered automatically. This architecture explains both the strengths and the limitations of the technology: it provides protection outside the hospital without implantation, but it protects only while it is worn, and it does not reproduce the full capabilities of an ICD, including chronic antitachycardia pacing or routine bradycardia pacing [7,9]. The initial WEARIT/BIROAD experience demonstrated that a wearable system could terminate malignant ventricular tachyarrhythmias in patients considered at high transient risk [10]. Subsequent national experience involving thousands of patients confirmed that clinically important VT/VF could be detected and treated, while also showing that outcomes were tightly coupled to wear time [11]. WEARIT-II then prospectively enrolled 2000 patients with a median LVEF of approximately 25%; median wear was 22.5 h/day for roughly 90 days, sustained ventricular tachyarrhythmias occurred during the protected interval, inappropriate therapy was uncommon, and many patients did not proceed to an ICD because LVEF improved [12]. Large German and European experiences reported similarly high median daily wear times in routine practice [13,14,15].

**Figure 1:**
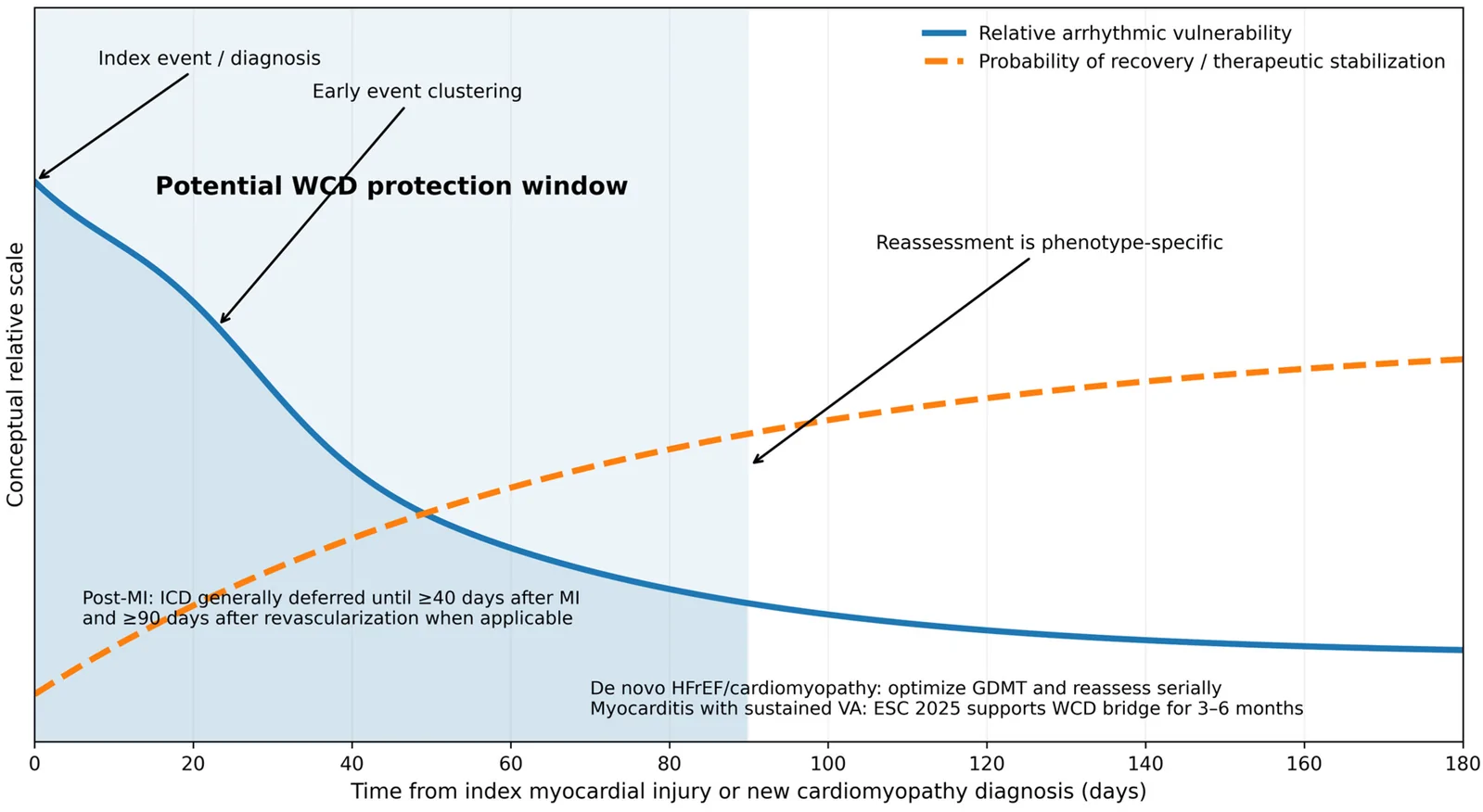
Conceptual temporal mismatch between early arrhythmic vulnerability and subsequent functional/therapeutic stabilization after an acute myocardial injury or a new diagnosis of cardiomyopathy. The WCD can provide temporary protection during a phenotype-specific reassessment window: post-MI ICD timing follows guideline waiting periods, de novo HFrEF recovery may continue beyond 90 days, and the 2025 ESC myocarditis guideline supports a 3–6-month WCD bridge after sustained ventricular arrhythmia. The curves are schematic and not derived from data.

An appropriate shock is an important clinical event and, when delivered for VF or unstable sustained VT, is a credible marker of a potentially lethal episode. Nevertheless, some treated arrhythmias might have self-terminated, and observational WCD cohorts preferentially include patients whom clinicians already judged to have sufficient risk and sufficient ability to use the device. Conversely, a trial based on assignment rather than actual wear can underestimate biologic efficacy when exposure is poor. The evidence must therefore be read through both lenses. The WCD is most rational when three conditions coexist: the immediate arrhythmic risk is clinically meaningful; the risk is expected to change over weeks or months; and an irreversible device decision can reasonably be deferred. In that setting, the WCD is neither an “early ICD” nor a substitute for optimal care. It is a temporary safety layer around revascularization, initiation and titration of guideline-directed medical therapy (GDMT), rhythm-control treatment, tissue healing, and reassessment (Figure 1).

## 4. Early Post-Myocardial Infarction

Severe left ventricular dysfunction, residual ischaemia, autonomic instability, myocardial edema, border-zone conduction heterogeneity, and evolving scar can all facilitate VT/VF. At the same time, the substrate and competing risks are changing rapidly. This biology explains why a low LVEF immediately after MI is prognostically important but is not equivalent to a stable indication for a permanent primary-prevention ICD. In the DINAMIT trial, prophylactic ICD implantation early after MI reduced arrhythmic death but did not reduce all-cause mortality because non-arrhythmic mortality increased [19]. IRIS trial reached a similar overall conclusion, demonstrating no survival advantage from early prophylactic ICD implantation despite the ability of the device to prevent some arrhythmic deaths [20]. Observational WCD studies nevertheless demonstrate that the waiting interval contains real ventricular arrhythmic events. Epstein et al. described 8453 early post-MI patients prescribed a WCD; 133 patients (1.6%) received appropriate shocks. Among treated patients, 75% experienced their first WCD-treated event within the first month and 96% within the first 3 months, with a median interval of 16 days from the index MI to WCD therapy [21]. Zishiri et al. reported a potential protective role after coronary revascularization in patients with left ventricular dysfunction during a period in which ICD implantation would generally be deferred [22]. Smaller European experiences also documented appropriate therapy in selected early post-MI patients [23]. The VEST trial remains the pivotal randomized evidence in this setting. A total of 2302 patients with acute MI and LVEF ≤ 35% were randomized in a 2:1 ratio to WCD plus guideline-directed therapy (*n* = 1524) or guideline-directed therapy alone (*n* = 778) [24]. The trial was initially funded by the National Institutes of Health with additional support from ZOLL Medical; after 2011, funding was provided exclusively by ZOLL Medical [24]. Median daily WCD use in the device group was 18.0 h (IQR, 3.8–22.7), with substantial between-patient variation and declining adherence over time. This was substantially lower than the median daily wear of 23.4 h/day reported in the WEARIT-France registry [14]. At 90 days, arrhythmic death, the primary endpoint, occurred in 1.6% of patients assigned to the WCD and 2.4% of controls (relative risk, 0.67; 95% CI, 0.37–1.21; *p* = 0.18), and therefore was not significantly reduced. All-cause mortality was 3.1% in the WCD group versus 4.9% in the control group (relative risk, 0.64; 95% CI, 0.43–0.98; uncorrected *p* = 0.04); because this was a secondary endpoint and the *p*-value was not adjusted for multiple comparisons, the finding should be interpreted cautiously. During the trial, 20 patients in the device group (1.3%) received at least one appropriate WCD shock, providing a useful absolute event proportion for comparison with observational WCD cohorts. Of the 48 patients who died in the device arm, only 12 were wearing the WCD at the time of death [24]. Subsequent as-treated and per-protocol analyses found stronger associations between greater WCD wear and lower arrhythmic and all-cause mortality [25]; however, these non-randomized exposure-based comparisons are susceptible to healthy-adherer bias and residual confounding, because patients who consistently wear the device may also differ in medication adherence, follow-up engagement, baseline frailty, and other prognostically relevant characteristics.

Real-world data after VEST have added context rather than eliminated the controversy. A single-centre study suggested that WCD-supported discharge after MI with left ventricular dysfunction can reduce hospital or coronary-care length of stay [26]. An Austrian cohort reported high median wear time and appropriate shocks in selected post-MI patients, but did not demonstrate a clear mortality advantage over historical randomized experience [27]. The French WICD-MI study provided a different type of information: among patients with early post-infarction left ventricular dysfunction who ultimately received an ICD, ventricular arrhythmia during WCD use strongly identified a subgroup with recurrent arrhythmias and higher subsequent mortality [28]. The German nationwide SCD-PROTECT study, whose data were derived exclusively from the manufacturer database and the ZOLL remote Patient Management System (ZPM), has substantially expanded the contemporary evidence base. Among 19,598 WCD-treated patients with newly diagnosed non-ischaemic cardiomyopathy or MI/coronary artery disease, the cumulative incidence of first appropriate WCD treatment during the observed WCD period was 1.3% overall, 1.1% in patients with NICM, and 1.5% in those with MI/CAD [29]. The 1.5% cumulative incidence observed in the MI/CAD subgroup is closely consistent with the 1.6% proportion of patients receiving appropriate shocks reported by Epstein et al. in an early post-MI population [21]. Mean WCD wear duration was 65.9 ± 43.8 days (median, 62 days [IQR, 34–89]). Importantly, information on the reason for WCD discontinuation was available for 9861 of the 19,598 patients; among these patients with documented end-of-use data, 5203 (52.8%) had LVEF improvement to >35% recorded [29]. Median daily wear was 23.4 h/day in the NICM group and 23.5 h/day in the MI/CAD group [29], substantially higher than the 18.0 h/day observed in VEST. The early risk appears several-fold higher than the stable chronic risk seen in many ICD populations, yet functional recovery during the same interval is common [29,30]. SCD-PROTECT is particularly valuable for quantifying event timing in the era of modern therapy and high adherence, but it remains an observational WCD-treated cohort without an untreated control; therefore, it cannot, by itself, establish a mortality benefit.

## 5. Newly Diagnosed Cardiomyopathy and HFrEF

Modern HFrEF therapy can produce substantial reverse remodeling, and the speed with which the four pharmacological pillars are initiated has shortened the time required to obtain prognostic benefit. Yet the first weeks remain a period in which the arrhythmic substrate may still be active, and the final need for an ICD is unknown [4]. The uncertainty surrounding primary-prevention ICD implantation in non-ischaemic cardiomyopathy is also informed by the DANISH trial. Among 1116 patients with symptomatic non-ischaemic systolic heart failure, prophylactic ICD implantation did not significantly reduce all-cause mortality compared with usual care (21.6% vs. 23.4%; hazard ratio, 0.87; 95% CI, 0.68–1.12; *p* = 0.28), although sudden cardiac death was significantly reduced (4.3% vs. 8.2%; hazard ratio, 0.50; 95% CI, 0.31–0.82; *p* = 0.005) [31].

Early observational studies demonstrated that the risk is not homogeneous by cardiomyopathy cause. In a decade-long single-centre experience, Singh et al. characterized WCD therapies in newly diagnosed ischaemic and non-ischaemic cardiomyopathy and showed that clinically relevant arrhythmias can occur during the period before reassessment [32]. The PROLONG strategy formalized the concept of protected therapeutic optimization: patients with newly diagnosed LVEF ≤ 35% received intensive HF therapy and WCD protection, and ICD decisions were delayed selectively when LVEF showed improvement or remained borderline [33]. A meaningful proportion of patients who would have met an ICD criterion at three months subsequently recovered further, while ventricular arrhythmias still occurred in a small subgroup. PROLONG-II showed that patients without an ICD indication after the protected optimization period had a favorable longer-term course, supporting the safety of a structured reassessment strategy rather than automatic implantation at the earliest possible date [34]. HF-OPT brought this concept into the contemporary GDMT era. Although 598 patients with de novo HFrEF entered the study phase, the serial LVEF analysis through Day 180 was restricted to 487 patients with LVEF measurements available at index, Day 90, and Day 180 [35]. Among these evaluable patients, 46% (95% CI, 41–50%) had improved to an LVEF > 35% by Day 90, increasing to 68% (95% CI, 63–72%) by Day 180. Longer-term echocardiographic follow-up was available in 392 patients, among whom 77% (95% CI, 72–81%) had an LVEF > 35% at Day 360. Sustained ventricular arrhythmias were concentrated during the first 90 days [35]. A small single-centre observational cohort of 41 WCD-treated patients illustrated how multiparametric follow-up can be integrated into a bridge-to-decision strategy rather than using the device solely for shock protection [36]. Over a mean follow-up of 62 ± 38 days, no WCD interventions occurred; at the end of follow-up, 15 of 41 patients (36.6%) still had an indication for ICD implantation, and 12 of 41 (29.3%) ultimately underwent implantation. A separate real-world analysis by Dell’Era et al. similarly suggested that a WCD-guided waiting strategy can improve selection for ICD implantation and may lower short-term costs compared with early implantation, although the sample was small and non-randomized [37].

The bridge-to-diagnosis concept is also relevant when the cardiomyopathy itself may be reversible. A 2026 real-world study of arrhythmia-induced cardiomyopathy in newly diagnosed HF patients using WCD protection showed substantial LVEF recovery after rhythm control and a marked reduction in subsequent ICD indications [38]. Peripartum cardiomyopathy provides another example: recovery is common, but a subset of women remains at early arrhythmic risk, and WCD observational data support temporary rather than reflexive permanent protection in selected cases [39,40]. Likewise, Takotsubo syndrome and other transient cardiomyopathies may occasionally present with high-risk ventricular arrhythmias, but absolute event rates are generally low and selection is critical. The 2026 meta-analysis focused specifically on non-ischaemic cardiomyopathy provides complementary evidence for interpreting the absolute arrhythmic event burden during WCD use [39]. Across 50 non-controlled observational studies including 10,066 patients, pooled appropriate shock proportions were 1% (87/7708; 95% CI, 1–2%) in generic newly diagnosed NICM, 2% (16/1049; 95% CI, 1–2%) in myocarditis, 3% (7/183; 95% CI, 0–20%) in peripartum cardiomyopathy, and 2% (2/102; 95% CI, 0–7%) in Takotsubo syndrome. Importantly, the approximately 1% pooled proportion observed in generic NICM is closely consistent with the 1.1% cumulative incidence of first appropriate WCD treatment reported in the NICM subgroup of SCD-PROTECT [29,39]. Thus, the apparent difference between these studies largely reflects the use of different denominators and reporting metrics rather than conflicting estimates of arrhythmic risk (Table 1).

## 6. Myocarditis

Ventricular arrhythmias in myocarditis may arise from edema, triggered activity, heterogeneous conduction, or scar. During active inflammation, polymorphic ventricular arrhythmias may be prominent; later, monomorphic VT may signal a more fixed scar-related substrate. This distinction is clinically important because LVEF can recover while arrhythmogenic scar persists. Observational WCD cohorts have demonstrated both the feasibility of temporary protection and the heterogeneity of risk. Tscholl et al. reported WCD detection and treatment experience in myocarditis, supporting its role during the healing phase [41]. Blaschke et al. likewise highlighted that suspected myocarditis is not a uniform low-risk diagnosis and that WCD prescription should be linked to the clinical and imaging phenotype rather than to the diagnostic label alone [42]. In an international multicentre registry, El-Battrawy et al. evaluated patients with myocarditis and reduced LVEF or ventricular tachyarrhythmia; LVEF improved substantially during follow-up, while arrhythmic events were concentrated in patients with higher-risk presentations [43]. A systematic review and meta-analysis of acute myocarditis presenting with arrhythmia further confirmed that an arrhythmic presentation identifies a subgroup with meaningful recurrent SCD/ventricular arrhythmia risk [44]. In the 2025 ESC guideline, “A WCD should be considered for 3–6 months in patients with sustained ventricular arrhythmia during the acute phase of myocarditis as a bridge to recovery” (Class IIa, level of evidence C) [6]. The guideline also emphasizes that sustained ventricular arrhythmia in acute myocarditis, although uncommon, has a substantial recurrence risk, with higher risk associated with features such as monomorphic VT, chronic active myocarditis, anteroseptal late gadolinium enhancement (LGE), and reduced LVEF. A persistent or extensive LGE pattern may indicate residual scar despite normalization of systolic function, and expert reviews have long emphasized the need to integrate imaging, rhythm history, and clinical phenotype when estimating SCD risk [45]. Although most of the strongest LGE outcome data derive from dilated cardiomyopathy rather than acute myocarditis, the principle is consistent. In non-ischaemic dilated cardiomyopathy, myocardial fibrosis detected by LGE is associated with mortality and SCD [46]; mid-wall LGE predicts SCD even in patients with only mild or moderate LV systolic impairment [47]; and meta-analysis shows a strong association between LGE and ventricular arrhythmia/SCD across a range of LVEF values [48]. A practical myocarditis pathway should distinguish at least four groups. First, uncomplicated myocarditis without ventricular arrhythmia, severe LV dysfunction, syncope, or high-risk scar generally does not justify routine WCD use. Second, myocarditis with severe but potentially reversible LV dysfunction may warrant temporary protection after individualized risk assessment. Third, sustained VT/VF during the acute phase is now a guideline-supported WCD indication when definitive ICD placement is deferred [6]. Fourth, myocarditis associated with a persistent malignant substrate, high-risk genetic disease, giant-cell myocarditis, cardiac sarcoidosis, recurrent sustained arrhythmia, or other features that establish a durable secondary-prevention indication should not be kept in an indefinite WCD holding pattern. In those patients, the WCD is, at most, a bridge to definitive therapy.

## 7. Risk Stratification Beyond LVEF

The history of SCD prevention has made LVEF indispensable, but the WCD literature exposes its limitations. LVEF is a marker of global pump function, not a direct measurement of scar architecture, electrical instability, autonomic tone, or transient inflammation. Moreover, in the clinical situations for which WCDs are prescribed, LVEF is explicitly expected to change. A risk model based entirely on the value that is most likely to change during follow-up is conceptually unstable. Contemporary WCD registry analyses increasingly seek phenotypic predictors. In an international multicentre study, Kreimer et al. evaluated predictors of ventricular tachyarrhythmia among WCD users and showed that clinically detectable features can enrich risk beyond a single ejection-fraction threshold [49]. SCD-PROTECT likewise demonstrated substantial early arrhythmic event rates in both ischaemic and non-ischaemic disease despite subsequent LVEF recovery in more than half of patients [29]. Conversely, a 2025 study of temporal LVEF trends before sudden death in HF underlined that systolic function can evolve and that a single remote measurement may poorly represent risk close to the event [50]. A more informative short-term framework considers substrate, trigger, timing, and trajectory. Substrate includes infarct scar, mid-wall fibrosis, inflammatory LGE, genetic cardiomyopathy, aneurysm, and severe persistent chamber remodeling. Trigger includes residual ischaemia, acute inflammation, electrolyte instability, rapid ventricular arrhythmia, or recurrent decompensation. Timing asks how recently the myocardial injury or HF diagnosis occurred; the 2025 meta-analysis found higher WCD intervention rates when mean follow-up was ≤60 days [16]. Trajectory incorporates whether LVEF, volumes, biomarkers, congestion, exercise capacity, and rhythm burden are improving, static, or worsening. This framework better matches the biological reason for prescribing a temporary device. Rhythm information deserves particular weight. Sustained VT/VF is qualitatively different from isolated premature ventricular complexes. Nonsustained VT, a high or changing ventricular ectopic burden, syncope suspected to be arrhythmic, and recurrent WCD-recorded events can all shift the balance toward prolonged protection or definitive ICD therapy. The WICD-MI study is an example of how an arrhythmia occurring during the WCD period can identify a subgroup with a markedly different subsequent trajectory [28]. In myocarditis, prior ventricular arrhythmia may be more predictive than LVEF alone [43,44,45]. The prognostic signal associated with CMR-defined fibrosis is substantial, although most supporting evidence derives from DCM populations not selected for WCD use. In non-ischaemic DCM, Gulati et al. found that midwall fibrosis was independently associated with SCD or aborted SCD (adjusted HR, 4.61; 95% CI, 2.75–7.74), while increasing fibrosis extent was also independently associated with arrhythmic risk (HR, 1.10 per unit increase; 95% CI, 1.05–1.16) [46]. Among patients with DCM and LVEF ≥40%, Halliday et al. reported SCD or aborted SCD in 17.8% of patients with midwall LGE versus 2.3% without LGE (HR, 9.2; 95% CI, 3.9–21.8), with an adjusted HR of 9.3 (95% CI, 3.9–22.3) [47]. In the meta-analysis by Di Marco et al., LGE was associated with ventricular arrhythmia or SCD even in studies with mean LVEF > 35% (OR, 5.2; 95% CI, 3.4–7.9), and the pooled adjusted association across studies reporting multivariable analyses was HR 6.7 (95% CI, 3.6–12.5) [48]. Within a proposed WCD bridge-to-decision framework, CMR assessment should focus on the presence, distribution, and extent of LGE; scar location and pattern (including mid-wall, subepicardial, or infarct-related scar); LV and RV volumes and systolic function; and, where inflammatory disease is suspected, myocardial edema and tissue-characterization parameters such as T1/T2 mapping and extracellular volume when available. The timing of CMR should be indication-specific: early imaging is most useful when it can characterize the substrate or establish an inflammatory diagnosis that may influence immediate risk assessment, whereas repeat imaging after the recovery or treatment interval may help determine whether edema has resolved, scar persists, and the substrate has become more or less arrhythmogenic. Importantly, neither a specific LGE percentage nor a mapping threshold has been prospectively validated to determine WCD prescription or discontinuation.

**Figure 2:**
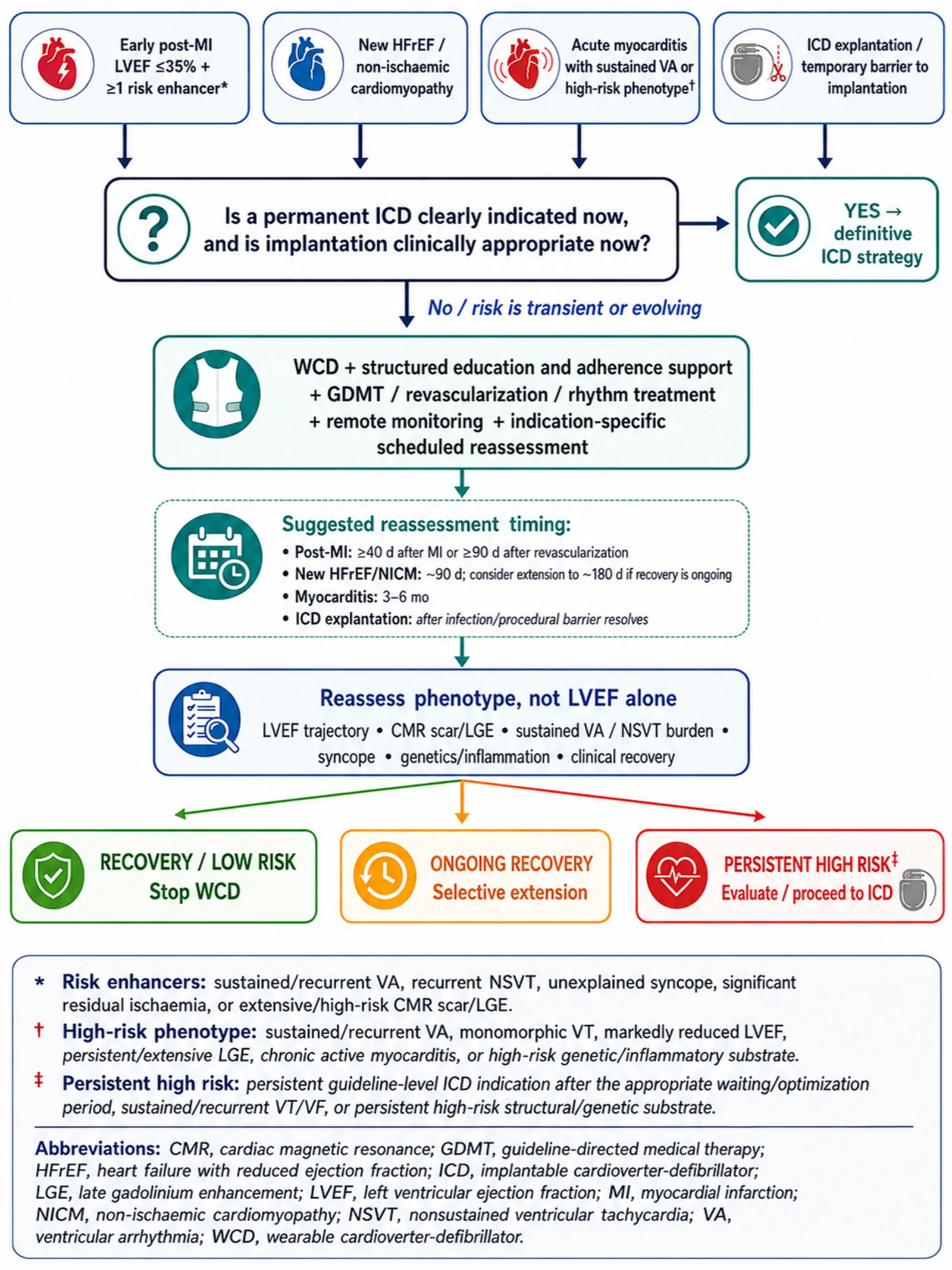
Proposed WCD bridge-to-decision framework for patients with transient or evolving arrhythmic risk. This framework is a proposal based on expert interpretation of the available evidence and has not been prospectively validated.

This multidimensional model also helps prevent overtreatment. A newly diagnosed patient with LVEF 34%, no scar, no ventricular arrhythmia, rapid improvement on therapy, and excellent clinical stability is not equivalent to a patient with LVEF 38%, extensive LGE, recurrent NSVT, and unexplained syncope. The latter may have a higher short-term arrhythmic risk despite a numerically better LVEF. WCD decisions should therefore be individualized around risk concentration and reversibility rather than around a single percentage (Figure 2).

## 8. Multiparametric Monitoring Platform, Adherence and Safety

Modern WCD systems can also provide information on physical activity [51]. A WCD-guided six-minute walk test performed at home can be feasible and reproducible [52]. Real-world HF programs have used heart-rate, wear-time, activity, and other device-derived trends as a telemonitoring layer [36,53,54]. However, there is no standardized, validated multiparametric WCD algorithm that has been shown in randomized trials to reduce HF hospitalization or mortality. The monitoring capability should therefore be viewed as a useful adjunct to a structured clinical pathway, not as a proven substitute for established HF remote-monitoring systems.

Structured adherence programs can improve wear time. Kellnar et al. showed that standardized training and adherence surveillance can increase daily use and help patients remain protected, although quality-of-life burdens remain relevant [55]. International registry analyses found broadly similar adherence between women and men [56], and age-related analyses suggest that older patients may wear the device at least as consistently as younger patients despite greater comorbidity [57,58]. Safety concerns are real but generally uncommon. Inappropriate shocks occur in a small minority of patients in modern registries and meta-analyses [16,17,18]. False alarms, skin irritation, pressure discomfort, sleep disruption, body-image concerns, and anxiety are more common and can erode adherence. A conscious patient’s ability to abort therapy is an important safety mechanism but also creates a requirement for adequate cognition and training. Patients who cannot reliably manage the device, who have no feasible caregiver support when needed, or who require continuous pacing are poor candidates.

## 9. Other High-Risk Transitional Scenarios

The clearest non-primary-prevention indication for a WCD is temporary loss of ICD protection. After extraction of an infected or malfunctioning ICD system, the patient may retain the same underlying secondary- or primary-prevention indication but require a period of infection treatment, wound healing, or procedural planning before reimplantation. National registry data show a significant early incidence of VT/VF after ICD explantation and support outpatient WCD protection during this interval [59]. More recent multicentre data similarly report appropriate WCD therapies after explant and a high subsequent reimplantation rate [60]. Earlier observational work also demonstrated the feasibility of outpatient protection after device removal [61]. Post-cardiac surgery is another heterogeneous temporary-risk scenario. A large multicentre cardiac-surgery cohort documented WCD use during periods of depressed LVEF and early postoperative recovery [62]. Some patients improve substantially after revascularization or valve surgery, whereas others remain at persistent risk and transition to an ICD. Patients awaiting transplantation, catheter ablation, or resolution of a reversible arrhythmic trigger are sometimes managed with a WCD, but the evidence is more limited and phenotype-specific. In such settings, the decision is best anchored in whether the patient has a credible short-term risk of treatable VT/VF, whether the underlying intervention is expected to change that risk, and whether an ICD is temporarily unsuitable.

## 10. Cost-Effectiveness and Health-System Impact

A device rented or reimbursed for several weeks or months may appear expensive compared with no device, but the relevant alternatives can include prolonged hospitalization, premature ICD implantation with future generator changes and complications, or death from an otherwise treatable early ventricular arrhythmia. Conversely, if baseline arrhythmic event risk is very low, if adherence is poor, or if the device is used for long periods without changing management, even technically successful therapy can be economically inefficient. The post-ICD-explant setting has produced some of the most favorable economic estimates. Healy and Carrillo modeled WCD use after removal of an infected ICD system and reported an incremental cost-effectiveness ratio of approximately USD 26,436 per quality-adjusted life-year (QALY), depending on assumptions about sudden-arrest risk and timing of reimplantation [63]. In Italy, Boriani et al. performed a cost-minimization analysis comparing WCD-supported outpatient management with hospitalization after ICD explantation and estimated a discounted direct saving of approximately EUR 1782 per patient [64]. These studies are intuitive because WCD therapy can replace inpatient monitoring during a temporary procedural barrier, rather than being added to otherwise routine outpatient care. Economic evaluation after MI is more controversial because it depends on the assumed mortality effect. An Italian health technology assessment used conservative intention-to-treat VEST inputs and estimated an incremental cost-effectiveness ratio of EUR 47,709/QALY, below the EUR 60,000/QALY threshold used in that analysis [65]. In England, a 2025 cost-utility model estimated GBP 23,024/QALY for WCD plus GDMT [66]. A 2025 Polish analysis estimated PLN 132,400/QALY (reported by the authors also as approximately EUR 31,100/QALY) [67]. A 2026 Spanish analysis estimated EUR 26,145/QALY and a 78.3% probability of cost-effectiveness at a EUR 30,000/QALY threshold [68]. Earlier US modeling reported an estimate of USD 60,600/QALY after MI [69] (Table 2).

Some recent models use as-treated or per-protocol VEST estimates, which generate a larger modeled survival benefit than the randomized intention-to-treat effect. Avoiding a premature ICD is another potential source of value. ICDs are highly effective when appropriately indicated, but implantation creates long-term device costs, generator replacements, follow-up requirements, inappropriate therapy risk, and procedural or infectious complications. Contemporary registry data suggest that real-world appropriate ICD therapy rates may be lower than those observed in older landmark trials in some primary-prevention populations [70]. Device infection can lead to hospitalization, extraction, reimplantation, morbidity, and substantial cost [71]. Contemporary studies also show that appropriate and inappropriate ICD shocks remain clinically important outcomes that vary with substrate and programming [72,73].

## 11. Guideline Synthesis and Practical Patient Selection

Guideline recommendations are deliberately conservative because the evidence differs by indication. The 2021 ESC HF guideline permits WCD use for selected patients with HF at risk of SCD for a limited period or as a bridge to an implanted device [4]. The 2022 ESC ventricular arrhythmia/SCD guideline recommends or permits WCD use in selected temporary-risk scenarios while emphasizing that routine early post-MI use is not supported [3]. The 2023 ANMCO position paper provides a pragmatic Italian framework for high transient-risk conditions, including early post-MI dysfunction, newly diagnosed HFrEF, myocarditis, and temporary removal of an implanted device [8]. The 2025 ACC/AHA ACS guideline retains a Class 2b, B-R statement that early post-MI WCD usefulness for improving survival is uncertain in LVEF ≤ 35% [5]. The most important recent change is the 2025 ESC myocarditis guideline, with a Class IIa, C recommendation [6] (Table 3).

Five questions should be answered before every prescription. First, is the estimated near-term risk of treatable VT/VF high enough to justify protection? Second, is that risk expected to change over the next 1–6 months? Third, is permanent ICD implantation currently inappropriate, premature, or temporarily contraindicated? Fourth, can the patient realistically achieve high daily wear time and manage alarms? Fifth, is there a scheduled reassessment plan with objective criteria for stopping the WCD, extending it, or proceeding to an ICD? If any of these questions have no credible answer, the prescription should be reconsidered.

Adherence should be treated as a clinical variable. Programs should include hands-on fitting, response-button training, management of skin irritation, clear bathing instructions, contact pathways for recurrent alarms, early review of wear-time data, and involvement of family or caregivers when appropriate. High adherence in European registries and in the SCD-PROTECT population shows that median daily wear times of 23.4 h/day in NICM and 23.5 h/day in MI/CAD are achievable in structured care [14,15,29]. International multicentre experience has also shown that high adherence and low inappropriate-therapy rates are achievable in real-world WCD practice [74]. Recent national experience from Poland supports implementation in routine clinical settings when organized pathways are present [75]. Conversely, a 2026 Japanese analysis highlighted a gap between guideline-based eligibility and actual WCD implementation after MI, illustrating that reimbursement, clinician familiarity, workflow, and patient acceptance influence real-world uptake [76].

## 12. Gaps in the Evidence and Future Directions

The principal gap remains comparative effectiveness. The field has one large randomized post-MI trial and a large volume of observational evidence. Registries are excellent for measuring actual wear time, shock incidence, device performance, rare safety events, and the clinical trajectory after prescription, but they cannot reliably answer what would have happened without the device. Future studies should therefore use indication-specific designs rather than pooling all WCD users into a single population. A second priority is an adherence-optimized randomized or pragmatic trial. VEST demonstrated the practical difficulty of testing a wearable therapy when assigned exposure is incomplete [24,25]. Future trials should incorporate structured education, remote adherence surveillance, rapid troubleshooting, and predefined strategies for patients who cannot tolerate the device. The estimand should be prespecified: intention-to-treat remains essential for policy effectiveness, but complementary exposure-adjusted analyses should be planned to distinguish device efficacy from implementation failure without overclaiming causality. Third, selection should move beyond LVEF. CMR scar quantification, LGE distribution, extracellular volume and mapping, rhythm burden, biomarkers, clinical trajectory, and selected genetic data could be integrated into short-term prediction models. The WCD itself can contribute continuous rhythm and activity information [35,50,51,52,53]. Machine-learning approaches may ultimately identify combinations of features that predict near-term VT/VF better than static thresholds, but such models require external validation and calibration across health systems. A model that predicts events in WCD-selected patients may not transport to unselected HF or post-MI populations because the original prescribing decision already introduces selection. Fourth, modern GDMT changes both numerator and denominator. Rapid initiation of angiotensin receptor-neprilysin inhibition, beta-blockers, mineralocorticoid receptor antagonists, and sodium-glucose cotransporter-2 inhibitors can accelerate reverse remodeling and reduce cardiovascular mortality [4,35]. As therapy improves, the average arrhythmic event rate may decline, potentially reducing the value of broad WCD prescription. At the same time, earlier discharge and shorter inpatient monitoring increase the need for reliable outpatient risk management. Economic models must therefore be continually updated rather than relying on event rates from historical cohorts. An additional priority is to determine whether findings generated predominantly with the established garment-based WCD platform are generalizable to newer device technologies. Alternative systems with different form factors and detection strategies are now available or under clinical evaluation. The garment-based ASSURE WCD (Kestra Medical Technologies) received FDA premarket approval in 2021 and has been evaluated in a 130-patient, 30-day study of alarm performance and in a separate evaluation of its defibrillation waveform [77,78]. The Jewel Patch WCD (Element Science) represents an adhesive-based approach intended for near-continuous wear, with first-in-human defibrillation results published in 2024 [79]. No head-to-head clinical comparison of these platforms, their detection algorithms, or alarm rates has yet been published. Accordingly, cross-study differences in alarm burden or device performance cannot be interpreted as evidence of comparative superiority between WCD systems.

Finally, patient-centered outcomes deserve more attention. Wearing a WCD for 20–23 h/day affects sleep, intimacy, clothing, exercise, anxiety, body image, and the perceived security of leaving hospital. The same device can be reassuring to one patient and highly burdensome to another. Shared decision-making should therefore include the uncertainty of survival benefit in specific indications, the expected duration of use, the practical burden, and the planned endpoint. A technically sophisticated strategy that a patient will not wear is not an effective strategy.

## 13. Limitations

This review has limitations. First, although the literature search covered PubMed, EMBASE, and the Cochrane Library from inception through July 2026 and used predefined clinical and methodological concepts, it was designed as a narrative state-of-the-art review rather than as a formal systematic review. Accordingly, study selection was not based on a prospectively registered protocol, and no PRISMA flow diagram or de novo risk-of-bias assessment was performed. Studies included in Table 1 were selected to represent the pivotal randomized trial, the largest contemporary registries, major prospective bridge-to-decision studies, and the most recent systematic reviews/meta-analyses informing the principal clinical indications discussed in the text. Second, additional primary studies were identified through reference chaining from major reviews and from the authors’ previous WCD publications. Although this approach can identify relevant studies not captured by database searches, it may also introduce citation bias by preferentially propagating previously cited bodies of evidence. Third, most of the major clinical evidence discussed in this review was generated using a single garment-based WCD platform. This concentration of evidence limits external validity, particularly for device-specific outcomes such as alarm burden, detection performance, adherence, and treatment delivery, and findings should not automatically be extrapolated to newer WCD systems with different architectures or algorithms.

## 14. Conclusions

The WCD has matured from a proof-of-concept external defibrillator into a clinically established, but still selectively indicated, tool for transient arrhythmic risk. The most consistent evidence across two decades is that the device can detect and terminate sustained ventricular tachyarrhythmias, that clinically relevant events cluster early after major myocardial injury or new cardiomyopathy diagnosis, and that many patients recover sufficiently to avoid a permanent ICD. The most important limitation is equally clear: technical efficacy and observational shock rates do not prove a population-level survival benefit. The optimal contemporary use of the WCD is consequently phenotype- and time-dependent, and the future of the WCD is therefore not broader use based on LVEF alone, but more precise use based on timing, CMR-defined substrate, rhythm phenotype, treatment trajectory, and patient-level ability to maintain exposure.

## Figures and Tables

**Table 1:** Contemporary evidence informing WCD use in post-MI and cardiomyopathy populations.

Study	Design/Sample	Setting	WCD Wear Time	Key Finding	Interpretation
WEARIT/BIROAD, 2004 [10]	Prospective/retrospective feasibility cohorts; *n* = 289	Mixed high-risk patients	≈93 days	Demonstrated ambulatory detection and termination of VT/VF	Established technical feasibility; not a survival trial
WEARIT-II, 2015 [12]	Prospective registry; *n* = 2000	Ischaemic, non-ischaemic and inherited disease	Median 90 days	Median wear 22.5 h/day; sustained VA during 90-day bridge; low inappropriate therapy	Supports protected risk stratification and frequent avoidance of ICD after recovery
VEST, 2018 [24]	Randomized trial; *n* = 2302	Post-MI, LVEF ≤ 35%	90-day intervention period; actual total days worn not reported as a single summary measure	Primary arrhythmic-death endpoint not significantly reduced: RR 0.67 (95% CI, 0.37–1.21; *p* = 0.18); median wear 18 h/day	Defines survival uncertainty for routine post-MI use
PROLONG, 2017 [33]	Prospective structured optimization cohort	New HFrEF, LVEF ≤ 35%	Mean 101 ± 89 days	Further LVEF recovery beyond the initial 3 months in selected patients; VA still occurred during bridge	Supports selective extension when recovery is ongoing
WEARIT-France, 2021 [14]	Prospective national cohort; *n* = 1157	Mixed transient-risk indications	Median 62 days (IQR, 37–97)	Median wear ~23.4 h/day; appropriate shocks with a low inappropriate-shock rate	Shows high adherence with standardized education/remote follow-up
HF-OPT, 2024 [35]	Prospective HF optimization study; *n* ≈ 600	De novo HFrEF	WCD prescribed for 90 ± 14 days during registry phase; continuation thereafter at physician discretion	LVEF > 35% in 46% (95% CI, 41–50%) at 90 d, 68% (95% CI, 63–72%) at 180 d, and 77% (95% CI, 72–81%) at 360 d among the respective evaluable populations	Reassessment beyond 90 d may be reasonable in selected improving patients
WICD-MI, 2024 [28]	French nationwide observational cohort	Early post-MI LV dysfunction; later ICD recipients	Median 22 days (IQR, 9–52) in VA+/WCD and 89 days (IQR, 56–122) in VA−/WCD	VA during WCD use strongly predicted recurrent VA and mortality	WCD-recorded arrhythmia can identify a persistent high-risk phenotype
Matteucci et al., 2025 [16]	Systematic review/meta-analysis; 40 studies; *n* = 59,647	Mixed WCD indications	Variable across included studies; study-specific wear duration, with analyses stratified by ≤60 vs. >60 days	Pooled appropriate intervention 3% (95% CI, 2–3%); prediction interval 1–8%; higher incidence with shorter follow-up	Average effect is heterogeneous; timing and selection are central
SCD-PROTECT, 2025 [29]	German nationwide observational study; *n* = 19,598	New NICM or MI/CAD	Mean 65.9 ± 43.8 days; median 62 days (IQR, 34–89)	First appropriate-treatment incidence rates: 6.10 (95% CI, 5.31–7.00) per 100 patient-years in NICM and 8.64 (95% CI, 7.41–10.05) in MI/CAD; cumulative incidence of first appropriate WCD treatment: 1.3% overall, 1.1% in NICM, and 1.5% in MI/CAD	Quantifies an early, front-loaded arrhythmic risk together with frequent functional recovery
Providência et al., 2026 [39]	Meta-analysis; 50 observational studies; *n* = 10,066 NICM	New NICM and subtypes	Variable across included studies; no single pooled WCD wear duration	Appropriate shocks: NICM 1% (95% CI, 1–2%), myocarditis 2% (1–2%), peripartum cardiomyopathy 3% (0–20%), Takotsubo 2% (0–7%)	Absolute rates are modest; indiscriminate use likely inefficient

**Table 2:** Selected WCD economic evaluations. Original study currencies are retained as the primary estimates.

Study	Health System/Scenario	Reported Result (Original Currency; USD Equivalent)	Price Year	Time Horizon	Key Limitation/Interpretation; Manufacturer Involvement
Healy & Carrillo, 2015 [63]	United States; after infected ICD removal	USD 26,436/QALY	2014 USD	5 years; WCD/reimplantation window 1–8 weeks	Sensitive to SCA risk, WCD efficacy and reimplantation timing. No ZOLL study funding was reported; Carrillo disclosed membership in the ZOLL speakers’ bureau.
Boriani et al., 2021 [64]	Italy; ICD explantation	COST-MINIMIZATION (not an ICER): EUR 1782 saving/patient vs. hospitalization (≈USD 2070)	2018 EUR	Lifetime; monthly cycles	Cost-minimization assumes equal effectiveness. Partially supported by an unrestricted ZOLL grant; some authors disclosed ZOLL speaker fees.
Botto et al., 2022 [65]	Italy; early post-MI	EUR 47,709/QALY (≈USD 55,409/QALY)	2018 EUR	Lifetime	Conservative VEST intention-to-treat inputs; Italian WTP threshold EUR 60,000/QALY. Partially supported by an unrestricted ZOLL grant; author ZOLL speaker fees disclosed.
Kontogiannis et al., 2025 [66]	England; early post-MI	GBP 23,024/QALY (≈USD 31,187/QALY)	2021/22 GBP	Lifetime	Uses adherence/exposure-dependent VEST effectiveness assumptions. Funded by ZOLL; commissioned analysts and ZOLL-employed coauthors disclosed.
Mitkowski et al., 2025 [67]	Poland; early post-MI	PLN 132,400/QALY (≈USD 35,613/QALY); authors also reported EUR 31,100/QALY	Mixed input years; NHF 2022 costs with selected items updated using 2024 price indices	Lifetime	Polish public-payer model; VEST as-treated inputs. Supported by an unrestricted ZOLL grant; several author ZOLL relationships disclosed.
González Costello et al., 2026 [68]	Spain; early post-MI	EUR 26,145/QALY (≈USD 30,365/QALY)	2025 EUR	Lifetime	Uses as-treated VEST estimates; Spanish WTP threshold EUR 30,000/QALY. Funded by ZOLL; commissioned analysts and ZOLL-employed coauthors disclosed.
Sanders et al., 2015 [69]	United States; early post-MI	USD 60,600/QALY	2014 USD	Lifetime	Highly sensitive to early SCA risk and assumed WCD effectiveness. Study supported by ZOLL; authors disclosed paid consultancy roles.

**Table 3:** Contemporary guideline synthesis and practical interpretation.

Clinical Scenario	Guideline Position	Practical Interpretation
Early post-MI, LVEF ≤ 35%	ACC/AHA ACS 2025: temporary WCD usefulness uncertain for survival, Class 2b, B-R [5]	Consider only after individualized enrichment of early arrhythmic risk; do not present routine WCD as proven survival therapy
New HFrEF/newly diagnosed cardiomyopathy	ESC HF 2021 and ESC VA/SCD 2022 permit WCD in selected limited-risk periods [3,4]	Use as a bridge to GDMT, rhythm treatment and serial reassessment when recovery is plausible
Acute myocarditis with sustained VA	ESC myocarditis 2025: consider WCD for 3–6 months as bridge to recovery, Class IIa, C [6]	Strongest new guideline development; integrate CMR, arrhythmia phenotype and genetic/inflammatory context
Temporary ICD contraindication/explantation	ESC/position statements support WCD when an ICD indication persists, but implantation is temporarily impossible [3,7,8]	High clinical coherence; set explicit reimplantation/infection-treatment plan
Any indication	No guideline substitutes WCD prescription for definitive reassessment	Define the stop/extend/ICD decision before discharge; monitor adherence continuously

## Data Availability

No new data were created or analyzed in this study. Data sharing is not applicable to this article.
